# Bio-guided bioactive profiling and HPLC-DAD fingerprinting of Ukrainian saffron (*Crocus sativus* stigmas): moving from correlation toward causation

**DOI:** 10.1186/s12906-021-03374-3

**Published:** 2021-07-21

**Authors:** Olha Mykhailenko, Vilma Petrikaitė, Michal Korinek, Mohamed El-Shazly, Bing-Hung Chen, Chia-Hung Yen, Chung-Fan Hsieh, Ivan Bezruk, Asta Dabrišiūtė, Liudas Ivanauskas, Victoriya Georgiyants, Tsong-Long Hwang

**Affiliations:** 1 Department of Pharmaceutical Chemistry, National University of Pharmacy of Ministry of Health of Ukraine, 4-Valentinivska st, Kharkiv, 61168 Ukraine; 2 Laboratory of Drug Targets Histopathology, Institute of Cardiology, Lithuanian University of Health Sciences, Sukilėlių pr. 13, LT-50162 Kaunas, Lithuania; 3 Institute of Biotechnology, Life Sciences Center, Vilnius University, Saulėtekio al. 7, LT-10257 Vilnius, Lithuania; 4 Graduate Institute of Natural Products, College of Pharmacy, Kaohsiung Medical University, Kaohsiung, 80708 Taiwan; 5 Department of Biotechnology, College of Life Science, Kaohsiung Medical University, Kaohsiung, 80708 Taiwan; 6 Graduate Institute of Natural Products, College of Medicine, Chang Gung University, Taoyuan, 33302 Taiwan; 7 Research Center for Chinese Herbal Medicine, Research Center for Food and Cosmetic Safety, and Graduate Institute of Health Industry Technology, College of Human Ecology, Chang Gung University of Science and Technology, Taoyuan, 33302 Taiwan; 8 Department of Pharmacognosy, Faculty of Pharmacy, Ain-Shams University, Organization of African Unity Street, Abassia, Cairo, 11566 Egypt; 9 Department of Pharmaceutical Biology, Faculty of Pharmacy and Biotechnology, German University in Cairo, Cairo, 11835 Egypt; 10 The Institute of Biomedical Sciences, National Sun Yat-sen University, Kaohsiung, 80424 Taiwan; 11 Department of Biochemistry and Molecular Biology, College of Medicine, Chang Gung University, Taoyuan, 33302 Taiwan; 12 Lithuanian University of Health Sciences, Department of Analytical and Toxicological Chemistry, A. Mickevičiaus g. 9, 44307 Kaunas, LT Lithuania; 13 Department of Anesthesiology, Chang Gung Memorial Hospital, Taoyuan, 33305 Taiwan; 14 Department of Chemical Engineering, Ming Chi University of Technology, New Taipei City, 24301 Taiwan

**Keywords:** *Crocus sativus*, Stigmas, Crude extract, HPLC, Cytotoxic activity

## Abstract

**Background:**

Saffron or stigmas of *Crocus sativus* L. is one of the most valuable food products with interesting health-promoting properties. *C. sativus* has been widely used as a coloring and flavoring agent. Stigmas secondary metabolites showed potent cytotoxic effects in previous reports.

**Methods:**

The present study investigated the chemical composition and the cytotoxic effect of Ukrainian saffron crude extracts and individual compounds against melanoma IGR39, triple-negative breast cancer MDA-MB-231, and glioblastoma U-87 cell lines in vitro using MTT assay. Several bioactivity in vitro assays were performed. The chemical profile of the water and hydroethanolic (70%, v/v) crude extracts of saffron stigmas was elucidated by HPLC-DAD analysis.

**Results:**

Seven compounds were identified including crocin, picrocrocin, safranal, rutin, apigenin, caffeic acid, ferulic acid. Crocin, picrocrocin, safranal, rutin, and apigenin were the major active constituents of Ukrainian *C. sativus* stigmas. The hydroethanolic extract significantly reduced the viability of MDA-MB-231 and IGR39 cells and the effect was more potent in comparison with the water extract. However, the water extract was almost 5.6 times more active against the U-87 cell line (EC_50_ of the water extract against U-87 was 0.15 ± 0.02 mg/mL, and EC_50_ of the hydroethanolic extract was 0.83 ± 0.03 mg/mL). The pure compounds, apigenin, and caffeic acid also showed high cytotoxic activity against breast cancer, melanoma, and glioblastoma cell lines. The screening of the biological activities of stigmas water extract (up to 100 μg/mL) including anti-allergic, anti-virus, anti-neuraminidase, and anti-inflammatory effects revealed its inhibitory activity against neuraminidase enzyme by 41%.

**Conclusions:**

The presented results revealed the qualitative and quantitative chemical composition and biological activity of *Crocus sativus* stigmas from Ukraine as a source of natural anticancer and neuraminidase inhibitory agents. The results of the extracts’ bioactivity suggested future potential applications of saffron as a natural remedy against several cancers.

## Background

Saffron (*C. sativus* stigmas) is one of the most expensive spices that is cultivated in a few countries around the world [1]. The peculiarity of this spice along with its unique color and taste attracted the attention of scientists to investigate its nutritional and therapeutic properties [1, 2]. Saffron was used as a medicinal plant long before its use as a spice [3]. The healing properties of saffron against many human diseases were documented by ancient Mediterranean, Persian, and Arabian civilizations. Since the beginning of the twentieth century, a plethora of studies investigated the chemical and therapeutic properties of saffron using modern analytical, pharmacological, and clinical techniques to confirm its traditional use [2, 3].

The main producing countries of *C. sativus* are Iran, Morocco, and Spain. Italy, India, and France produce fewer quantities [2, 3]. The cultivation area of saffron is limited by environmental factors that affect its ontogenesis. Since 2015, Ukrainian farmers started the widespread cultivation of saffron for food-grade purposes [4]. Climatic conditions including temperature, humidity, light radiation, altitude, and soil conditions are different across Ukraine. These differences may affect the chemical composition and consequently the pharmacological properties of *C. sativus* harvested from different areas in Ukraine. Saffron is gaining popularity in Ukraine not only as a spice but as a medicinal raw material in folk medicine for eye diseases, diabetes, acute respiratory viral diseases, and cancers [5, 6]. According to the international standards (ISO/Technical Specification 3632), saffron stigmas from Ukraine are considered of high quality [4]. However, a complete chemical analysis of saffron cultivated in Ukraine has not yet been performed.

Cancer is one of the most devastating diseases that usually requires treatment with surgery and chemotherapy. These chemotherapeutics are known for dreadful effects on the quality of life and resistance triggering properties [7]. Therefore, there is a continuing need to search for alternative treatments from natural sources to decrease the dependence on chemical therapeutic agents and reduce chemotherapeutics’ toxic side effects. Among various uses of saffron, it is also considered a potential antitumor natural remedy [3]. The literature contains encouraging data on the antitumor activity of various saffron extracts and their components [8, 9], thus our attention was drawn to investigate the effects against some of the less-studied cancer cell lines.

The objective of this study was to compare the phytochemical content of the water and hydroethanolic (70%, v/v) crude extracts of *C. sativus* stigmas. The cytotoxic activity of the extracts and several individual compounds of saffron stigmas was evaluated against human melanoma IGR39, triple-negative breast cancer MDA-MB-231, and glioblastoma U-87 cell lines. Additionally, various bioactivities including antiallergic, anti-inflammatory, anti-viral, or anti-neuraminidase bioactivities of the water extract were assessed.

## Methods

### Reagents and chemicals

Acetonitrile and methanol were of HPLC grade and purchased from Roth GmbH (Karlsruhe, Germany). Reference compounds (crocin, safranal, apigenin, rutin, caffeic acid, ferulic acid, chlorogenic acid, gallic acid) were purchased from ChromaDex (Santa Ana, CA), Sigma-Aldrich (Saint Louis, MO), HWI Analytik GmbH, and Roth GmbH (Karlsruhe, Germany).

### Plant material

*Crocus sativus* L. (*Iridaceae* family) flowers were collected from the plantation in the village Lyubimivka (Kherson region, Ukraine) in November 2019 in accordance with the WHO Guidelines on Good Agricultural and Collection Practices (GACP) [10, 11]. The permission for harvest was obtained from the farmers according to the cooperation agreement. The raw material was collected and identified by Dr. Mykhailenko and the identification was verified by Dr. Gamulya (V.M. Karazin Kharkiv National University, Kharkiv, Ukraine). A specimen was deposited at the Herbarium of V.M. Karazin Kharkiv National University, Ukraine (CWN, voucher specimen No. CWN0056541). The flowers were collected manually, then the stigmas (saffron) were separated and dried for 2–3 h at 50 °C under forced air. Dried stigmas were stored in dark glass jars at 4 °C.

### Preparation of *C. sativus* stigmas extracts

*Water extract of C. sativus stigmas.* Saffron was ground in a mortar using a pestle. 5 g of stigmas powder was macerated with hot distilled water (500 ml, 80 °C) [12, 13], kept in a dark place for 24 h, then the extract was filtered. Maceration was repeated 2 more times with the residue under the same conditions. The resulting mixtures were combined, filtered, dried on a rotary evaporator at 80 °C.

*C. sativus* stigmas hydroethanolic (70%, v/v) extract was obtained with the same above method using ethanol/water 70/30 (v/v) (5 g, 500 mL) instead of water as the extracting solvent.

### Sample preparation for analysis

The dried extract (water and hydroethanolic, the weight of each sample was 0.01 g) was extracted with 10 mL of methanol using an ultrasonic bath at room temperature (20 ± 2 °C) for 30 min. The solution was filtered using a membrane filter (0.45 μm) before use. An aliquot of 10 μL was injected into the HPLC system for analysis. A standard solution was prepared by dissolving reference compounds including crocin, safranal, rutin, caffeic acid, chlorogenic acid, gallic acid, ferulic acid, and apigenin in methanol (1.0 mg/mL). These solutions were used for calibration. All samples were kept at 4 °C before use.

### HPLC conditions

Chromatographic separation of compounds was carried out using an ACE C_18_ column (250 mm × 4.6 mm, 5.0 μm; Pennsylvania, USA). Elution was performed at a flow rate of 1 mL/min. The binary solvent system of the mobile phase consists of solvent A (0.1% acetic acid in water) and solvent B (acetonitrile). All solvents were filtered through a 0.23 μm membrane filter after ultrasonic degassing. A gradient elution was applied: 0 min – 95% A and 5% B, 7 min – 95% A and 5% B, 67 min – 0% A and 100% B, 69 min – 95% A and 5% B, 75 min – 95% A and 5% B. The column temperature was constant at 25 °C. The injection volume of the sample solution was 10 μL. The standard solutions including crocin, safranal, rutin, apigenin, caffeic acid, chlorogenic acid, gallic acid, and ferulic acid were used for the calibration of a standard curve using an external standard method. The picrocorcin content in the extracts was recalculated as safranal equivalent. Analysis was performed in duplicate. Validation of the HPLC method was performed according to certain parameters [14] including specificity, linearity, precision, the limit of detection (LOD), and limit of quantitation (LOQ) (Tables 1 and 2).

**Table 1:** Calibration curves of the quantified reference standard compounds

Compound		Calibration curve^а^	Correlation coefficient ***r***^**2**^ (***n =*** 6)	Linear range (μg/mL)	RSD, %	LOD^b^ (ng/mL)	LOQ^c^ (ng/mL)
1	Caffeic acid	y = 57,646.8x-3853.48	0.9999218	0.72–91.92	1.56	20	60
2	Ferulic acid	y = 54,955.4x - 638.345	0.9999592	0.44–56.5	1.60	30	80
3	Rutin	y = 16,072.5x + 1499.73	0.9998787	0.16–20.24	1.07	96	290
4	Crocin	y = 3789.03x + 220.836	0.999588	1.15–147.2	1.28	100	300
5	Apigenin	y = 50,138.3x + 5722.97	0.9998899	0.2–25.76	0.53	25	80
6	Safranal	y = 39,230.1x-11,887.2	0.999529	1.33–42.56	1.35	120	360
7	Gallic acid	y = 32,880.6x-612.983	0.9999718	0.48–61.08	1.31	30	100
8	Chlorogenic acid	y = 29,930.2x-538.361	0.9999502	0.36–46	1.29	20	70

Note: ^a^concentration of compound (mg/mL); y, peak area; ^b^LOD, limit of detection (S/*N* = 3); ^c^LOQ, limit of quantification (S/*N* = 10)

**Table 2:** Precision and repeatability of the quantified compounds

Compound		Concentrate (μg/mL)	Precision				Repeatability	
			Intra-Day (***n*** = 3)		Inter-Day (***n*** = 3)		Recovery (%)	RSD (%)
			RSD (%)	Accuracy (%)	RSD (%)	Accuracy (%)		
1	Caffeic acid	11.49	1.05	102.02	0.52	98.49	100.01	0.46
		45.96	1.08	98.78	0.67	99.73	99.39	0.99
		91.92	0.64	100.35	0.95	98.17	100.17	0.37
2	Ferulic acid	7.06	0.68	100.22	0.90	98.29	99.11	0.69
		28.25	0.93	98.20	0.29	99.31	99.60	0.57
		56.5	1.22	100.24	0.46	98.28	100.12	0.49
3	Rutin	2.53	1.26	100.35	0.62	100.15	100.18	0.55
		10.12	1.29	101.12	0.80	99.21	100.65	0.92
		20.24	0.76	99.56	1.14	100.94	99.78	0.31
4	Crocin	1.23	0.87	102.5	0.80	101.36	101.07	0.85
		18.4	1.25	99.19	0.91	100.37	98.99	1.06
		73.6	1.22	98.97	0.81	100.27	100.29	0.98
5	Apigenin	4	0.74	100.75	0.57	98.76	100.56	0.79
		16	0.89	100.89	0.29	98.62	98.96	0.71
		32	0.88	100.70	0.70	98.03	99.80	1.02
6	Safranal	1.33	0.57	101.53	0.67	101.07	100.27	0.65
		10.64	0.78	98.68	0.53	99.14	99.58	0.77
		42.56	1.02	100.47	0.86	100.12	99.14	1.04
7	Gallic acid	7.65	0.57	99.81	0.75	101.37	101.07	0.65
		30.35	0.78	99.56	0.24	102.14	99.69	0.56
		61.20	1.02	101.53	0.38	101.32	100.09	0.94
8	Chlorogenic acid	5.75	1.31	101.12	0.38	98.40	100.69	0.86
		23	0.42	99.08	0.73	99.43	99.58	1.05
		46	0.96	100.27	0.48	98.24	101.91	0.97

### Apparatus

Liquid chromatography separation was performed using the Shimadzu Nexera X2 LC-30 AD HPLC system (Shimadzu, Japan) formed of a quaternary pump, an on-line degasser, a column temperature controller, the SIL-30 AC autosampler (Shimadzu, Japan); the CTO-20 AC thermostat (Shimadzu, Japan), SPD-M20A diode array detector (DAD). Ultrasonic Cleaner Set as used for ultra-sonication (Wise Clean WUC-A06H, Witeg Labortechnik GmbH, Germany), рН-meter – Knick Electronic Battery-operated pH Meter 911 PH (Portamess, Germany), rotary evaporator (Heidolph 2 WB eco, Laborata400 efficient, Germany).

### Data analysis

All data processing was carried out using LabSolutions Analysis Data System (Shimadzu Corporation). Statistical analysis was performed by one-way analysis of variance (ANOVA) followed by Tukey’s multiple comparison test with the software package Prism v.5.04 (GraphPad Software Inc., La Jolla, CA, USA). A *p*-value < 0.05 was considered significant.

### Cell culture

Human melanoma cancer cell line IGR39, human triple-negative breast cancer cell line MDA-MB-231, and human glioblastoma U-87 cell lines were obtained from the American Type Culture Collection (ATCC, Manassas, VA, USA). Cells were grown in DMEM Glutamax medium (Gibco, Carlsbad, CA, USA) containing 10% fetal bovine serum and 1% antibiotic mixture (10,000 U/mL penicillin and 10 mg/mL streptomycin; Gibco). All cells were incubated at 37 °C in a humidified atmosphere containing 5% CO_2_.

### Cell viability assay

The cells were treated with saffron extracts and their viability was determined by the MTT assay (Sigma-Aldrich Co.) as described elsewhere [15]. The cells were exposed to various concentrations of the tested extracts (from 1 mg/mL to 31.25 μg/mL), and after measurement of formazan solution absorbance, EC_50_ (half-maximal effective concentration of a drug/extract at which 50% of its maximum response is observed) values were calculated.

### Antiallergic activity in RBL-2H3 cells

A methylthiazole tetrazolium (MTT) assay was used to measure the possible toxic effects of the samples on RBL-2H3 cells as previously described [16]. The maximally tolerated dose of DMSO was 0.5%, not affecting RBL-2H3 cell growth. Triton X- 100 (0.5% solution) was used as the positive control causing the death of all cells in a well. The water stigmas extract was then subjected to an anti-allergic degranulation assay based on *β*-hexosaminidase release in RBL-2H3 mast cells induced by calcium ionophore (A23187) or antigen (IgE plus DNP-BSA) according to the previous methodology [17, 18]. Briefly, RBL-2H3 cells seeded in the 96-wells plate (2 × 10^4^ cells/well, A23187-induced assay) or 48-wells plate (3 × 10^4^ cells/well, antigen-induced assay) at 37 °C in 5% CO_2_ atmosphere for at least 5 h. then they were treated with samples or medium (untreated control) for 20 h. Cells were stimulated by the addition of calcium ionophore A23187 (1 μM) or cross-linking antigen DNP-BSA (100 ng/mL) to previously sensitized cells with anti-DNP IgE (0.1 μg/mL). After 1 h of incubation, the unstimulated cells were either lysed with 0.5% Triton X-100 solution for the total amount of *β*-hexosaminidase release or left untreated for the spontaneous release of *β*-hexosaminidase. Then aliquots of the wells’ supernatants (50 μL) were incubated with an equal volume (50 μL) of 1 μM of p-NAG (*p*-nitrophenyl-*N*-acetyl-*β*-D-glucosaminide, in 0.1 M citrate buffer, pH 4.5) serving as the substrate for the released *β*-hexosaminidase. After 1 h of incubation at 37 °C, the reaction was quenched by the addition of 100 μL of stop buffer (0.1 M Na_2_/NaHCO_3_, pH 10.0). Absorbance was measured at 405 nm on a microplate reader and the percentage inhibition of *β*-hexosaminidase release was calculated.

### Anti-inflammatory activity in human neutrophils

Blood was taken from healthy human donors using a protocol approved by the Chang Gung Memorial Hospital review board. Neutrophils were isolated following the standard procedure [19]. The inhibition of superoxide anion generation (respiratory burst) was measured based on ferricytochrome *c* reduction as previously described [20]. Briefly, preheated neutrophils (6 × 10^5^ cells·mL^− 1^) and 0.5 mg/mL ferricytochrome *c* solution were treated with the tested compounds or DMSO (control) for 5 min, and activated with formyl-methionyl-leucyl-phenylalanine (fMLF, 100 nM)/cytochalasin B (CB, 1 μg/mL) for 10 min. The absorbance was continuously monitored at 550 nm using Hitachi U-3010 spectrophotometer with constant stirring (Hitachi Inc., Tokyo, Japan). Calculations were based on the differences in absorbance with and without superoxide dismutase (SOD, 100 U/mL) divided by the extinction coefficient for the reduction of ferricytochrome *c* (ε = 21.1/mM/10 mm). Elastase release (i.e., degranulation from azurophilic granules) was evaluated as described before [21]. Briefly, neutrophils were equilibrated with elastase substrate, MeO-Suc-Ala-Ala-Pro-Val-p-nitroanilide (100 μM), at 37 °C for 2 min and then incubated with the sample for 5 min. Cells were activated by 100 nM fMLF and 0.5 μg/mL CB, and changes in the absorbance at 405 nm corresponding to elastase release were continuously monitored. The results were expressed as the percent of the initial rate of elastase release in the fMLF/CB-activated drug-free control system.

### Lipid droplet assay

Lipid droplet assay was performed according to a previous method using a BSA-conjugated oleic acid system in Huh7 cells as described previously [22]. Briefly, cells seeded in μClear® 96-wells plates (Greiner Bio-ONE, Frickenhausen, Germany) were treated with oleic acid and the tested sample or DMSO for 18 h. Paraformaldehyde was used to fix the cells, which were stained with 2 μg/mL Hoechst 33342 and 1 μg/mL BODIPY® 493/503. High Content Imaging (HCS) instrument was used to take and analyze images of the nuclei and lipid droplets (ImageXpress Micro System, Molecular Devices, Sunnyvale, CA, USA). The diameter settings were 8–25 μm for the nuclei and 0.5–2 μm for the lipid droplets.

### NRF2 activity

Nuclear transcription factor NRF2 activity was evaluated in HacaT normal cells and Huh7 cancer cells according to a previous methodology [23]. The cell line HaCaT/ARE (antioxidant response element) was developed using a HaCaT stable cell line carrying a fragment derived from pGL4.37[luc2P/ARE/Hygro] plasmid and the luciferase reporter gene luc2P. The reporter cells were cultured in Dulbecco’s Modified Eagle’s Medium (DMEM) (Gibco BRL, Grand Island, NY, USA) supplemented with penicillin (100 U/mL), streptomycin (100 μg/mL), 10% heat-inactivated fetal bovine serum (HyClone, Logan, UT, USA), and 100 μg/mL hygromycin at 37 °C in 5% CO_2_. The cells were seeded (1 × 10^4^ cells/well) in a 96-wells plate and treated with the sample for 18 h (single measurement). Resazurin (Cayman Chemical, Ann Arbor, MI, USA, final concentration of 0.1 mg/mL) was added and the cells were incubated for an additional 4 h at 37 °C. Fluorescence of the reduced resazurin in the supernatant of the cells (ex/em: 530 nm/590 nm) was detected using a Synergy HT Multi-Mode Reader (BioTek, Winooski, VT, USA) to determine cell viability. The cells were then harvested, and luciferase activity was measured according to the manufacturer’s protocol (Promega Corporation, Madison, WI, USA). The luciferase activity was normalized to cell viability.

### Protective against influenza and enterovirus

The anti-viral assay was performed by cytopathic effects of the extracts on the cells infected by influenza H1N1 [24], and enterovirus D68 [25]. Briefly, the 96-well tissue culture plates were seeded with MDCK cells (2 × 10^4^ per well) or RD cells (2 × 10^4^ cells /well) in E10 medium (DMEM containing 10% FBS, 100 U/mL penicillin (Gibco, USA), 100 μg/mL streptomycin (Gibco, USA), 2 mM L-glutamine (L-glutamine) (Gibco, Brazil), 0.1 mM nonessential amino acid mixture (NEAA, Gibco, USA) and incubated under 5% CO_2_ for 16–24 h at 37 °C. Then, the culture medium was withdrawn and the wells were washed once with Dulbecco’s phosphate-buffered saline (DPBS). The cells were infected with influenza virus (A/WSN/33) or enteroviruses at a nine-fold median tissue culture infective dose, with or without the addition of the samples. The treated cells were further incubated for 72 h at 37 °C. After 72 h, the medium was removed, and the cells were fixed with 4% paraformaldehyde for 1 h at room temperature. Then, 0.1% crystal violet was used to stain the cells for 20 min at room temperature. The cells density was measured by using a VICTOR3™ multilabel plate reader (PerkinElmer).

### Neuraminidase activity assay

A baculovirus displayed neuraminidase NA9 on the surface (NA9-Bac) as a pseudotyped influenza virus was used to evaluate the neuraminidase activity. An appropriate virus load of NA9-Bac was added into a 96-well plate and incubated with the extracts or compounds for 20 min at 37 °C. Then, each well was supplemented with 25 μL of diluted fluorescent MUNANA substrate. After incubation for 30 min at ambient temperature, 150 μL of stop solution was added. The fluorescence intensity was immediately detected using Synergy HT Multi-Mode Microplate Reader (BioTek). Zanamivir, a known neuraminidase inhibitor, was used as a positive control in this assay.

## Results and discussion

According to the reported data, the cytotoxic properties against different cancer cell lines were found for both the water and hydroethanolic extracts of *C. sativus* stigmas [1–3, 7, 9]. However, the research on the cytotoxic properties of *Crocus* spp. extracts against melanoma IGR39, triple-negative breast cancer MDA-MB-231, and glioblastoma U-87 cell lines is still lacking. Therefore, we studied the chemical composition and cytotoxic activity of Ukrainian saffron crude extracts against these cell lines, in addition to other bioactivities screening, to understand the potential applications of this medicinal plant.

### HPLC method validation

A validation study was conducted to demonstrate the applicability of the developed analytical method. The validation was done in terms of specificity, linearity, LOD, LOQ, precision, and recovery according to the International Conference on Harmonization guidelines [14]. The results are summarized in Tables 1 and 2. The regression equation for each reference standard compound, together with the LOD and LOQ values, are shown in Table 1. All the calibration curves showed acceptable linear regression (*r*^2^ ≥ 0.999). The overall intraday and interday precision RSDs were not more than 2.0%. The overall stability over 24 h and repeatability were not more than 2.0% for both parameters. The developed analytical method showed excellent precision with overall recovery in the range from 98 to 101% (RSD ≤ 2.0%) for all compounds. Therefore, the method was precise, accurate, and sensitive enough for the simultaneous quantitative evaluation of all compounds in *C. sativus* extracts.

The specificity is the ability of a method to discriminate between the study analytes and other constituents in the sample. Specificity was demonstrated by the separation of the analytes from other interfering compounds. The determination of the main compounds in the tested solutions was done by comparing the retention times of the peaks and UV-spectrum with those of the standard solution. The results showed that the conditions for the fingerprint analysis were repeatable and precise.

### Qualitative and quantitative analysis of the compounds

**Fig. 1:**
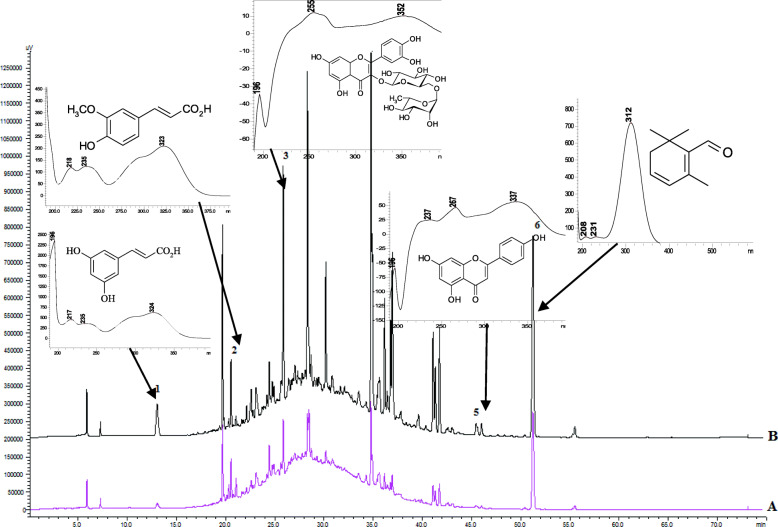
HPLC-DAD chromatograms of *C. sativus* stigmas water (**A** pink line) and hydroethanolic (70%, v/v) (**B** black line) crude extracts: caffeic acid (**1**); ferulic acid (**2**); rutin (**3**); apigenin (**5**); safranal (**6**). The detection wavelength was set at 310 nm

**Fig. 2:**
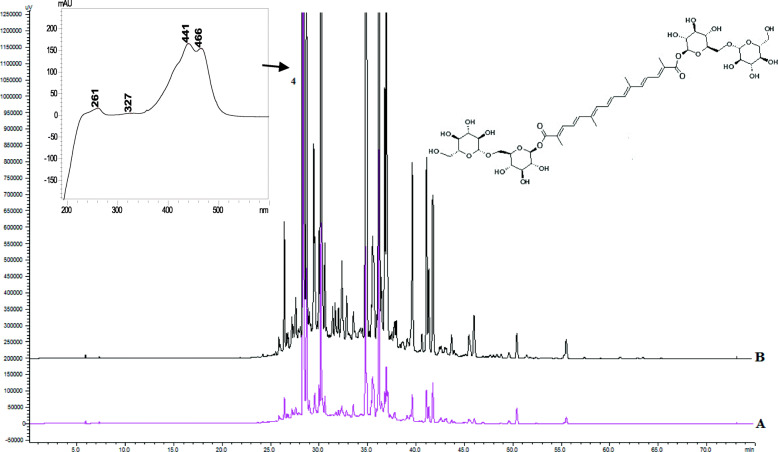
HPLC-DAD chromatograms of *C. sativus* stigmas water (**A** pink line) and hydroethanolic (70%, v/v) (**B** black line) crude extracts: crocin (**4**). The detection wavelength was set at 440 nm

**Fig. 3:**
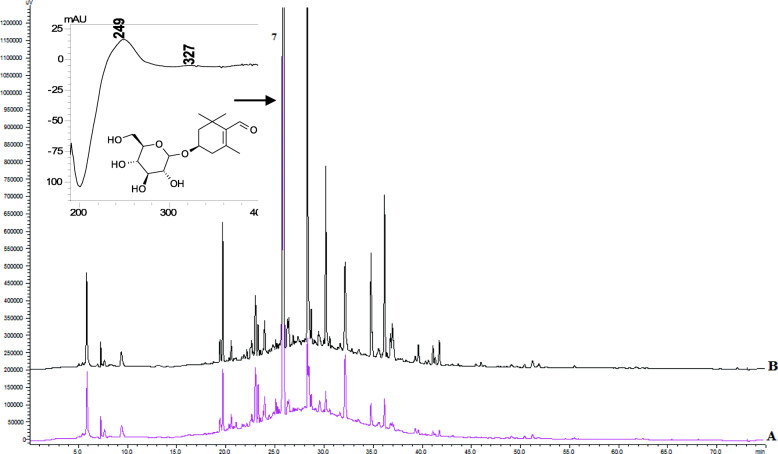
HPLC-DAD chromatograms of *C. sativus* stigmas water (**A** pink line) and hydroethanolic (70%, v/v) (**B** black line) crude extracts: picrocrocin. The detection wavelength was set at 250 nm

The extraction of the biologically active components from *C. sativus* stigmas was carried out under optimal conditions by maceration at room temperature in the dark to minimize the decomposition of the phenolic compounds and carotenoids [26]. To identify the composition of the active ingredients in the tested extracts, an HPLC-DAD method was used. The HPLC chromatograms of *Crocus* water and hydroethanolic (70%, v/v) crude extracts are shown in Figs. 1, 2 and 3. The determination of the compounds in the tested extracts was done by comparing the peak retention times and the UV spectra obtained from the chromatogram of the standard solution. The results of the components of the analysis of the crude extracts of Ukrainian *C. sativus* stigmas are presented in Table 3. Among the main and species-specific compounds in *C. sativus* stigmas extracts, the presence of crocin, safranal, and picrocrocin was analyzed. According to the published data, the quantitative content of crocin in the dried *C. sativus* stigmas from Italy, Greece, France, Spain varied depending on the growing conditions and processing methods. It ranged from 6 to 16% up to 30% [27].

**Table 3:** The content of biological active compounds (mg/g dry weight) in *Crocus sativus* stigmas water and hydroethanolic (70%, v/v) crude extracts

Compounds	RT, min	λ, nm	UV, λ _**max**_, nm	Stigmas extract	
				Water	Hydroethanolic
**Specific compounds**					
Crocin	28.37	440	261, 440, 466	38.27 ± 0.03	163.02 ± 1.16
Picrocrocin	25.88	250	249, 327	62.25 ± 0.10	197.19 ± 5.60
Safranal	55.85	310	231, 312	10.81 ± 0.03	146.66 ± 3.07
**Flavonoids**					
Rutin	22.48	310	256, 352	3.07 ± 0.02	14.81 ± 0.41
Apigenin	47.90	250	237, 267, 337	0.96 ± 0.01	8.39 ± 0.15
**Hydroxycinnamic acids**					
Caffeic acid	14.18	310	217, 236, 342	0.11 ± 0.00	0.38 ± 0.01
Ferulic acid	21.64	310	218, 236, 323	0.13 ± 0.03	0.26 ± 0.00

In the current study, the content of crocin in Ukrainian saffron was 38 mg/g (3.8%) in the water and 163 mg/g (16.3%) in the hydroethanolic extract, respectively (Table 3). For comparison, the content of crocin (identified as *trans*-crocetin bis(*β*-D-gentiobiosyl) ester) in the methanol extracts of the Iranian and Azerbaijan saffron was 45.99 and 48.47 mg/g, respectively, and for the alcoholic extract from the Spanish saffron, the content was 11.95 mg/g [28]. Thus, the content of crocin in Ukrainian samples was at least 2–3 times higher. The high content of secondary metabolites in the Ukrainian saffron extracts might be due to concomitant factors including the location of the cultivation site, altitude, soil type, climate, quality of planting material, irrigation periods, and harvest time. Previously, we investigated the chemical composition of *C. sativus* stigmas from different regions of Ukraine and the results showed high content of crocin, picrocrocin, and safranal in the raw materials [4].

The content of picrocrocin was recalculated as a safranal equivalent and was detected in the hydroethanolic extract reaching the maximum limit described in the literature of 197 mg/g (19.7%). Picrocrocin was detected in a lower concentration in the water extract (62 mg/g). In previous studies, the content of picrocrocin was determined in the range of 7–16% in saffron samples [29]. The content of picrocrocin in saffron methanol extract from Azerbaijan was only 26.93 mg/g. This value was obtained using Waters HPLC system, a Spherisorb ODS2 column (250 × 4.6 mm internal diameter) and the used mobile phase was a linear gradient of methanol in water from 10 to 100% containing 15% acetonitrile [28]. In the current analysis, we applied the Shimadzu system and ACE C_18_ column (250 mm × 4.6 mm, 5.0 μm). The selected gradient system of mobile phases consisted of solvent A (0.1% acetic acid in water) and solvent B (acetonitrile). This system provided the best peak separation. The selection of 257, 330, and 440 nm as the detection wavelengths resulted in an acceptable response and allowed the detection of all three major compounds (crocin, picrocrocin, and safranal), phenolic acids, and flavonoids. The column temperature was maintained at 25 °C throughout the analysis. HPLC fingerprints for *C. sativus* stigmas extracts were developed.

In the studied crude extracts of Ukrainian saffron, the safranal content was 146.6 mg/g in the hydroethanolic and 10.81 mg/g in the stigmas water extract. In previous studies, the content of safranal in saffron raw materials from Spain was 6 mg/g [27, 30], and for the Iranian saffron, the values were 0.07–0.29 mg/g [31]. The analysis of ethanol, water, and methanol-water extracts of saffron stigmas from Saudi Arabia indicated the presence of lower concentrations of crocin (10–16 mg/g) and safranal (5 mg/g) in comparison with our results [32].

In addition to the esters of crocetin, picrocrocin, and safranal, other biologically active compounds were identified in the Ukrainian saffron extracts. The current research presents the first data on the identification of flavonoid apigenin and rutin in *C. sativus* stigmas. It should be noted that the content of rutin and apigenin in the hydroethanolic *C. sativus* stigmas extract was significantly higher (14.8 mg/g and 8.38 mg/g, respectively) than in the water extract (3.07 mg/g and 0.96 mg/g).

According to the literature data, different phenolic acids such as caffeic, chlorogenic, and gallic were identified in the *C. sativus* stigmas [1]. Ferulic acid was only found in *C. cancellatus* subsp*. damascenus* stigmas [33]. However, in the crude extracts of Ukrainian saffron, chlorogenic and gallic acids were not identified. Ferulic and caffeic acids were found in the hydroethanolic extract of the Ukrainian stigmas extracts at 0.26 and 0.38 mg/g, respectively. In conclusion, the content of all detected constituents in the hydroethanolic extract was much higher than in the water extract.

The quantitative analysis of the biologically active compounds in the crude extracts showed that croсin (identified as *trans*-crocetin bis(*β*-D-gentiobiosyl) ester), picrocrocin, and safranal were the major components of *C. sativus* stigmas extracts in agreement with the literature data [27–32]. Previous studies highlighted the importance of these compounds for the biological activities of saffron [2, 3, 8, 9]. In addition to these compounds, the presence of flavonoids with their antiproliferative and cytotoxic activities [34] encouraged us to evaluate the cytotoxic activity of Ukrainian saffron extracts.

### Cytotoxic activity of saffron extracts

**Fig. 4:**
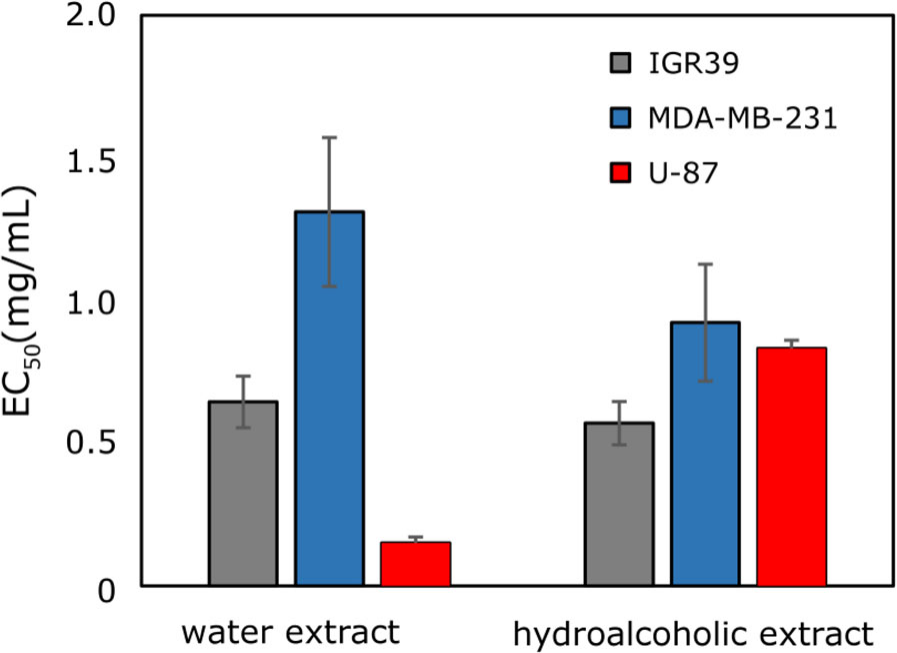
Cytotoxic activity of the extract against the tested cancer cell lines

The in vitro cytotoxic activity of saffron extracts was investigated against human melanoma IGR39, triple-negative breast cancer MDA-MB-231, and glioblastoma U-87 cell lines (Fig. 4). Hydroethanolic (70%, v/v) crude extract of *C. sativus* stigmas significantly reduced the viability of breast cancer and melanoma cells in comparison with the water extract. However, the water extract was about 5.6 times more active against the glioblastoma cell line (EC_50_ of the water extract against U-87 was 0.15 ± 0.02 mg/mL, and EC_50_ of the hydroethanolic extract was 0.83 ± 0.03 mg/mL). The higher activity of *Crocus* water extract against glioblastoma cell line might be due to the presence of hydrophilic biologically active compounds, such as amino acids, polysaccharides, carboxylic acids [3, 35]. In correlation with our results, several studies on plants such as *Inula helenium* [36], *Usnea longissimi* [37], and *Tragopogon porrifolius* [38] showed that water extracts exhibited higher activity against human U-87 glioblastoma compared with different ethanolic extracts. For instance, the published data [39] indicated that crocetin, a metabolite of major saffron component crocin, exhibited pronounced antitumor properties against U251, U87MG, U373, and U138 glioma cell lines. However, the saffron extract activity against the brain cancer cell line (U-87 cell line) was not reported before.

In other studies, saffron extracts showed anti-proliferative activity against several types of cancer cell lines at higher concentrations. For example, *C. sativus* stigmas methanolic extract demonstrated anti-proliferative activity against acute lymphoblastic leukemia cells (Jurkat cell line) (EC_50_ = 71 ± 2.50 μg/mL) [40]. *C. sativus* stigmas aqueous extract showed a dose-dependent inhibitory effect on the growth of human transitional cell carcinoma (TCC 5637) and mouse fibroblast (L929) cell lines at concentrations ranging from 400 μg/mL to 800 μg/mL [41]. Also, the stigmas aqueous extract exhibited a potent dose-dependent antineoplastic effect on highly metastatic murine B16-F10 melanoma cell line (40.7 to 73.6%, at 250 to 1000 μg/ml, 72 h treatment) [1]. In another study, the cytotoxic effect of saffron stigmas ethanolic extract was evaluated in HepG2 and HeLa cell lines (IC_50_ 950 and 800 μg/mL, respectively, 48 h) [42]. Saffron extract inhibited the proliferation of HCT-116 cells by 54.5% at a concentration of 1 mg/mL [43]. According to Abd Razak et al. (2017) [44], saffron extract and its main components could affect carcinogenesis in different models in vitro and in vivo.

The saffron demonstrated also other activities related to protection against cancer including radical scavenging activity, anti-mutagenic and immunomodulatory effects [45, 46].

### Cytotoxic activity of saffron individual compounds

**Fig. 5:**
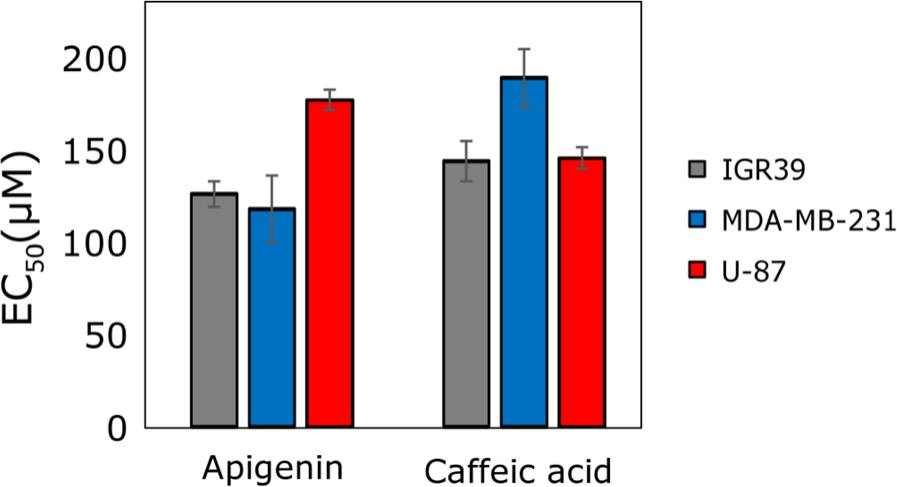
Anticancer activity of the tested active compounds against different cancer cell lines

To better understand how the activity could be related to the chemical composition, we evaluated the EC_50_ values for several major ingredients of saffron extracts (Fig. 5). According to the results of the current study, the most active substances against the tested breast cancer, melanoma, and glioblastoma cell lines were apigenin and caffeic acid with EC_50_ values ranging from 123.4 to 197.6 μM. Other substances (crocin and rutin) did not show cell viability reducing activity even at the highest tested concentrations (up to 1 mM, data not shown). The lack of activity of crocin and rutin in our study may be also associated with the specificity towards the selected cell lines. Previous studies on human and animal cancer cell lines demonstrated the cytotoxic activity of saffron as well as its main constituents (crocins, crocetin, safranal, picrocrocin) against leukemia, carcinoma, sarcoma, stomach, liver, prostate, cervix, ovary, breast, skin, lung, and colorectal cancer cell models, often using high concentrations [2, 8, 47]. For instance, the antiproliferative effects of crocin against several cancer and non-cancer cell lines were reported, however, a very high concentration was needed to reach an EC_50_ value [48–50]. There is an evidence that high concentrations of crocin (0.625–10 mg/mL) significantly inhibited HL-60 cell proliferation [48], as well as dose-dependently induced apoptosis and cell cycle arrest at the G2/M phase in MDA-MB-231 cells (approx. IC_50_ 5 mg/mL, 48 h) [49]. The authors, however, studied only the effect of an individual compound, crocin, but did not investigate the cytotoxic activity of saffron stigmas whole extract. The cytotoxic bioactivity of crocin and its metabolite crocetin was compared in lung A549, cervical HeLa, ovarian SK-OV-3, colorectal HCT-116, liver HepG2 cell lines [50]. Crocetin (EC_50_ = 0.16–0.61 mmol/L), showed 5- to 18-folds higher cytotoxicity than crocin (EC_50_ = 2.0–5.5 mmol/L) [50] and crocetin further inhibited proliferation glycolytic cancer cell lines A549 and HeLa (IC_50_ 0.11 mM for both cell lines) as well as lactate dehydrogenase (LDH) [51].

Several studies indicated that *C. sativus* extracts exhibited their cytotoxic effect due to the presence of not only crocin, picrocrocin, safranal, but also a plethora of phenolic compounds.

Apigenin is a common dietary flavonoid that showed the highest activity against melanoma IGR39 and breast cancer MDA-MB-231 cell lines (EC_50_ values were 131.8 ± 7.2 μM and 123.4 ± 19.0 μM, respectively) in our experiment (Fig. 5). The activity against the U-87 cell line was lower compared with caffeic acid. The results obtained were consistent with the previously described data [52, 53]. Apigenin reduced MDA-MB-231 cell viability at a similar concentration as in our experiments (12, 27, 42, and 49% inhibition at 25 μM, 50 μM, 75 μM and 100 μM, respectively) [54]. Apigenin also showed potent antiproliferative effect against human melanoma A375 cell line (EC_50_ was 33.02 μM) [52]. This activity was higher compared to that against IGR39 cells in our experiments which could be explained by compound specificity against different cell lines. Previous literature showed the antitumor efficacy of apigenin against several types of cancer in vitro and in vivo [55]. For instance, apigenin showed antiproliferative properties against the glioblastoma U1242 and U87 cells [56], and human melanoma A375 and C8161 cells in a concentration- and time-dependent manner [57]. Regarding the molecular basis of its activity, apigenin inhibited cancer cell proliferation by triggering cell apoptosis, inducing autophagy, decreasing cancer cell motility, migration, and invasion [55], and regulating immune response [58]. Multiple signaling pathways were modulated by apigenin, including PI3K/AKT, MAPK/ERK, JAK/STAT, NF-κB, and Wnt/*β*-catenin [55].

In our experiment, caffeic acid showed a similar level of activity against the IGR39 and U-87 cell lines (EC_50_ values were 150.5 ± 11.4 μM and 152.3 ± 6.2 μM, respectively), and a slightly lower against the MDA-MB-231 cell line (EC_50_ was 197.6 ± 16.3 μM). According to the literature, caffeic acid reduced cell viability against the MCF-7 cell line (EC_50_ 159 μg/mL) [59] and against the HCT15 cell line (approx. EC_50_ 800 μM) in time and dose-dependent manner [60]. Caffeic acid previously demonstrated cytotoxic activity against SK-Mel-28 human melanoma [61] as well as hepatocellular carcinoma, preventing the exaggerated formation of ROS [62]. It promoted the death of tumor cells through DNA oxidation, as well as angiogenesis reduction of VEGF-induced vascularization and the suppression of MMP-2 and MMP-9 expression, acting as antioxidant and pro-oxidant at the same time [62].

Rutin is a flavonol that demonstrated several pharmacological activities, including antioxidant, cytoprotective, vasoprotective, anticarcinogenic, neuroprotective, and cardioprotective activities [63]. According to different studies, rutin could cause a significant reduction in tumor size justifying its antileukemic potential [64]. Rutin is also known to inhibit cancer cell growth by cell cycle arrest and/or apoptosis along with the inhibition of proliferation, angiogenesis, and/or metastasis in colorectal cell lines [63]. However, in our study rutin did not show a substantial cell growth inhibition towards IGR39, MDA-MB-231, or U-87 cells at concentration up to 1 mM (data not shown).

Previous literature data indicated that saffron extracts and their constituents, crocin, crocetin, and safranal, apigenin or caffeic acid exhibited a selective toxic effect against cancer cells while toxicity against normal cells was negligible in vitro [56, 62, 65, 66] or in vivo [67, 68]. The molecular mechanisms of saffron extract and its active components are not yet fully understood, and further studies are needed to justify the use of saffron extracts in cancer treatment.

### Bioactivity screening and anti-neuraminidase activity

For the preliminary bioactivity analysis, the water extract of *C. sativus* stigmas was selected aiming to use the most eco-friendly solvent. There is a lack of studies on the anti-neuraminidase, anti-inflammatory, and antiviral activity of the stigmas extracts. According to the results (Table 4), the water extract (100 μg/mL) inhibited neuraminidase enzymatic activity by 41.0% in comparison with the positive control, zanamivir (97.4% at 1 μM). Phenolic compounds including apigenin, rutin, and caffeic acid were detected in Ukrainian saffron. Previous studies indicated that plant extracts rich in phenolic content inhibited the enzymatic activity of viral neuraminidase [69]. Also, the amino acid composition of the plant extracts was shown to determine the activity against influenza A virus neuraminidase [70, 71]. In our previous investigation, we studied the composition of amino acids in the water extract of *C. sativus* stigmas and found high content of amino acids including tyrosine (326.6 μg/g), methionine (84 μg/g), and alanine (60 μg/g) [72]. The stigmas extract was inactive in the other bioactivity tests (Table 4), including the anti-allergic (degranulation assay, 100 μg/mL), anti-viral (influenza H1N1 and enterovirus D68, 50 μg/mL), anti-inflammatory (respiratory burst and degranulation, 10 μg/mL), NRF2 expression in normal and cancer cell line (100 μg/mL), and lipid droplets assay (100 μg/mL). Our results together with the literature data suggested that the high content of amino acids as well as the presence of phenolic compounds may correlate with the neuraminidase inhibitory effects of *C. sativus* stigmas water extract, while its higher concentration might be required for the other bioactivities.

**Table 4:** The bioactivity evaluation results of saffron water extract, including the NRF2, neuraminidase, lipid droplets, anti-inflammatory, antiallergic activity, anti-influenza and anti-enterovirus activity

Sample	Relative NRF2 activity^a^ in HacaT cell^b^ (%, mean ± SD)	Relative NRF2 activity^a^ in Huh7 cell^b^ (%, mean ± SD)	Neuraminidase inhibition activity^c^ (%, mean ± SD)	Lipid droplet inhibition activity^d^ (%, mean ± SD)	Superoxide anion generation, human neutrophils^e^ (%, mean ± SEM)	Elastase release, human neutrophils^e^ (%, mean ± SEM)	A23187-induced degranulation assay, RBL-2H3 cells^f^ (%, mean ± SD)	Antigen-induced degranulation assay, RBL-2H3 cells^f^ (%, mean ± SD)	Protective activity against influenza H1N1, MDCK cells^g^	Protective activity against enterovirus 68, RD cells^g^
Saffron stigmas (extr. H_2_O)	172.8	132.1	41.0 ± 5.8	108.7 ± 14.3	9.0 ± 1.9**	5.8 ± 1.3*	5.0 ± 4.6	20.3 ± 4.2	inactive	inactive
TBHQ^h^	684.3 ± 53.3	–	–	–	–	–	–	–	–	–
Luteolin^i^	–	23.8 ± 0.3	–	–	–	–	–	–	–	–
Zanamivir^j^	–	–	96.8 ± 0.2	–	–	–	–	–	–	–
TC^k^	–	–	–	9.1 ± 0.8	–	–	–	–	–	–

^a^Relative luciferase activity (NRF2) was calculated by normalizing luciferase activity to cell viability and presented as the fold to solvent control. Saffron stigmas 100 μg/mL

^b^HacaT, a normal skin cell line. Huh7, a liver cancer cell line

^c^Neuraminidase inhibition assay. Saffron stigmas 100 μg/mL

^d^Lipid droplet count, the average LD counts/cell of OA were used as a standard for 100% of fatty loading in the Huh7 cell line. Saffron stigmas 100 μg/mL

^e^Results are presented as mean ± SEM (n = 3) compared with the control (fMLF/CB), *P < 0.05 and **P < 0.01. Genistein served as the positive control and inhibited 99.7%

of superoxide anion generation at 10 μg/mL and 101.2 of elastase release at 30 μg/mL [23]. Saffron stigmas 10 μg/mL

^f^The cytotoxicity of the sample was evaluated by MTT assay (95.0 ± 8.7%). Inhibition of *β*-hexosaminidase release was evaluated and the results are presented as mean ± SD (*n* = 3) compared to the untreated control (DMSO). Dexamethasone (10 nM) was used as a positive control and inhibited 65.7 and 66.3% of A23187- and antigen-induced *β*-hexosaminidase release, respectively [23] Saffron stigmas 100 μg/mL

^g^The protective effects were evaluated based on the viability of cells infected by the virus. Saffron stigmas 50 μg/mL. Inactive, no significant inhibition

^h^TBHQ, 2-(1,1-dimethylethyl)-1,4-benzenediol, was used as positive control for Nrf2 activation. Drug concentration is 10 μM

^i^Luteolin, was used as a negative control for Nrf2 activation. The drug concentration is 50 μM

^j^Zanamivir, was used as a positive control for neuraminidase inhibition. The drug concentration is 1 μM

^k^TC, Triacsin C, is an inhibitor of long fatty acyl CoA synthetase and was used as a positive control for lipid droplet inhibition. The drug concentration is 1 μM

## Conclusion

**Fig. 6:**
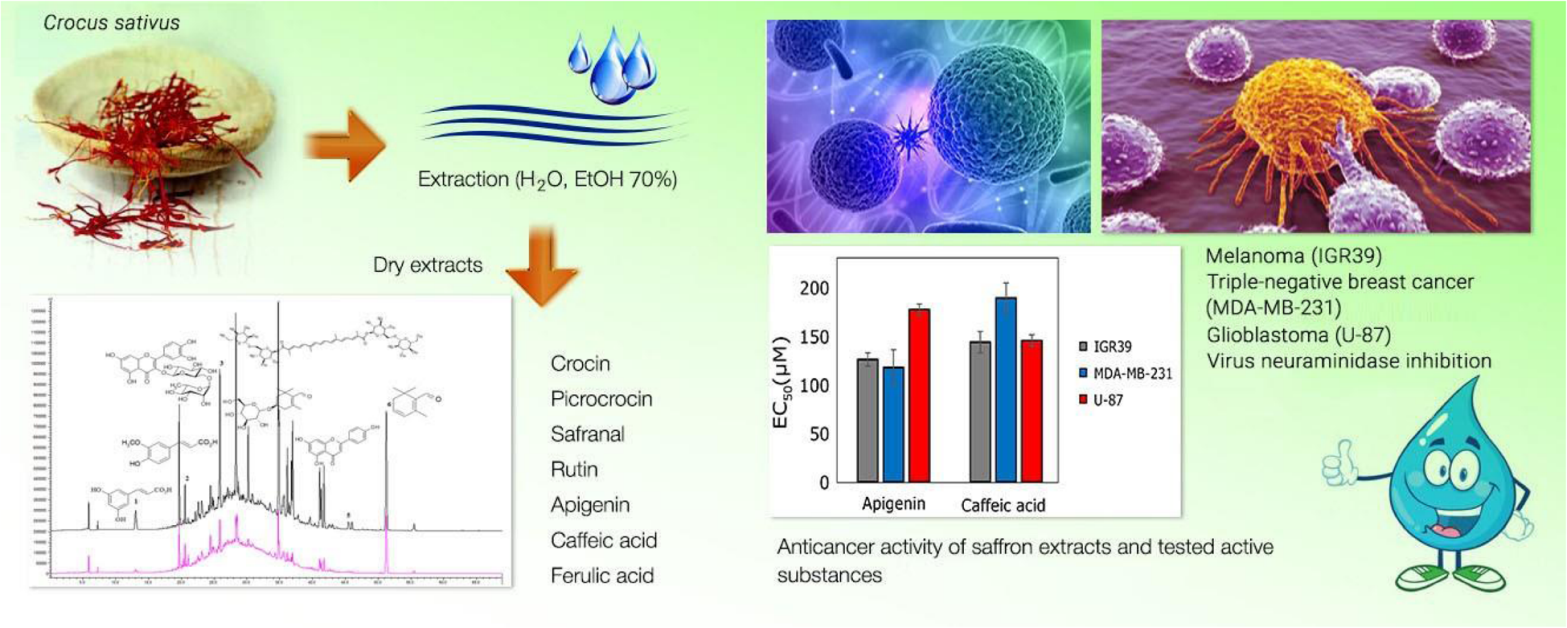
Overview of the study results on phytochemical analysis and antitumor and other bioactivities of Ukrainian saffron. Antitumor activity against melanoma, triple-negative breast cancer, and glioblastoma cell lines was evaluated in saffron extracts as well as individual compounds

The present study evaluated the cytotoxic activity of *Crocus sativus* stigmas from Ukraine and correlated results with its major constituents, as identified by HPLC analysis (Fig. 6). The water and hydroethanolic (70%, v/v) extracts exhibited a cytotoxic effect against melanoma IGR39, triple-negative breast cancer MDA-MB-231, and glioblastoma U-87 cell lines. The water extract of saffron stigmas possessed higher activity than the hydroethanolic extract against U-87 cell lines. This study also described the HPLC method for the qualitative analysis and quantitative determination of apocarotenoids, flavonoids, and phenol carboxylic acids in *C. sativus* stigmas extracts. Moreover, rutin, apigenin, and ferulic acid were identified in *C. sativus* stigmas for the first time. Apigenin and caffeic acid showed activity on the selected cancer lines. The results of the current study indicated the need for further research to determine the mechanisms responsible for the established anti-cancer activity.

## Data Availability

All the materials will be available for research purposes upon request from the corresponding author.

## Abbreviations

HPLC: High performance liquid chromatography
MTT assay: Assessing cell metabolic activity
EC_50_: Half maximal effective concentration
CB: cytochalasin B
DMEM: Dulbecco’s modified Eagle’s medium
DMSO: Dimethyl sulfoxide
DNP-BSA: Dinitrophenyl-conjugated bovine serum albumin
FBS: Fetal bovine serum
fMLF: formyl-methionyl-leucyl-phenylalanine
HPLC-DAD: High-performance liquid chromatography coupled with diode array detector
NRF2: Nuclear factor erythroid 2-related factor 2
RBL: Rat basophilic leukemia
